# Phenylalanine-Rich Peptides Potently Bind ESAT6, a Virulence Determinant of *Mycobacterium tuberculosis*, and Concurrently Affect the Pathogen's Growth

**DOI:** 10.1371/journal.pone.0007615

**Published:** 2009-11-10

**Authors:** Krishan Kumar, Megha Tharad, Swetha Ganapathy, Geeta Ram, Azeet Narayan, Jameel Ahmad Khan, Rana Pratap, Anamika Ghosh, Sachin Kumar Samuchiwal, Sushil Kumar, Kuhulika Bhalla, Deepti Gupta, Krishnamurthy Natarajan, Yogendra Singh, Anand Ranganathan

**Affiliations:** 1 Recombinant Gene Products Group, International Centre for Genetic Engineering and Biotechnology, New Delhi, India; 2 Institute of Genomics and Integrative Biology, Delhi, India; 3 Immunology Group, International Centre for Genetic Engineering and Biotechnology, New Delhi, India; 4 School of Life Sciences, Jawahar Lal Nehru University, New Delhi, India; University of Hyderabad, India

## Abstract

**Background:**

The secretory proteins of Mycobacterium tuberculosis (*M. tuberculosis*) have been known to be involved in the virulence, pathogenesis as well as proliferation of the pathogen. Among this set, many proteins have been hypothesized to play a critical role at the genesis of the onset of infection, the primary site of which is invariably the human lung.

**Methodology/Principal Findings:**

During our efforts to isolate potential binding partners of key secretory proteins of *M. tuberculosis* from a human lung protein library, we isolated peptides that strongly bound the virulence determinant protein Esat6. All peptides were less than fifty amino acids in length and the binding was confirmed by *in vivo* as well as *in vitro* studies. Curiously, we found all three binders to be unusually rich in phenylalanine, with one of the three peptides a short fragment of the human cytochrome c oxidase-3 (Cox-3). The most accessible of the three binders, named Hcl1, was shown also to bind to the *Mycobacterium smegmatis* (*M. smegmatis*) Esat6 homologue. Expression of *hcl1* in *M. tuberculosis* H37Rv led to considerable reduction in growth. Microarray analysis showed that Hcl1 affects a host of key cellular pathways in *M. tuberculosis*. In a macrophage infection model, the sets expressing *hcl1* were shown to clear off *M. tuberculosis* in much greater numbers than those infected macrophages wherein the *M. tuberculosis* was not expressing the peptide. Transmission electron microscopy studies of *hcl1* expressing *M. tuberculosis* showed prominent expulsion of cellular material into the matrix, hinting at cell wall damage.

**Conclusions/Significance:**

While the debilitating effects of Hcl1 on *M. tuberculosis* are unrelated and not because of the peptide's binding to Esat6–as the latter is not an essential protein of *M. tuberculosis*–nonetheless, further studies with this peptide, as well as a closer inspection of the microarray data may shed important light on the suitability of such small phenylalanine-rich peptides as potential drug-like molecules against this pathogen.

## Introduction

Tuberculosis (TB), a disease caused by *M. tuberculosis*, is completely curable, and yet, two million succumb to it every year [1], [2]. In India, that along with sub-Saharan Africa has the largest number of TB cases, partial adherence to directly observed drug treatment regimen, coupled with non-availability of the drugs in remote areas combine devastatingly to exacerbate the problem, resulting in multi-drug resistant strains that then *de facto* necessitate the scientific community's search for newer anti-TB molecules [3]–[5]. Tied with this seemingly intractable predicament is the lengthy anti-TB therapy that lasts on average six to eight months, giving ample opportunity for poor patients to play truant [6]. The search for new drug-like molecules against TB that can reduce the length of therapy as well as address the problem of resistance is, therefore, an urgent one.

As a starting point for our own efforts towards addressing this urgency, we decided to identify potent protein-protein interactions that must take place between the secretory proteins of *M. tuberculosis*, the carrier of TB, and their human counterparts at the primary site of infection, the human lung, in order for the infection to either take root, or, as in many cases, be cleared [7]. Our aim was, once such interactions had been identified, to use *de novo* protein/peptide libraries and screen for entities that are able to disrupt such interactions [8], [9]. That some *M. tuberculosis* proteins, mostly those that are found in the culture filtrate of *M. tuberculosis*–and thus termed ‘the culture-filtrate’ proteins or CFPs–that many among this set interact with human lung proteins has been known for some time [10]–[14]. A few of such interactions have also been studied in detail [14]–[16]; however, no comprehensive picture of the infection process has as yet emerged.

Here, we report our investigations with one such secretory protein, the Esat6 protein that has been implicated in the virulence and pathogenesis of *M. tuberculosis* [17]–[20]. Briefly, Esat6 is a 100 aa long protein that normally exists, indeed is secreted, as a 1∶1 complex with its cohort the Cfp10 protein, the latter being also of roughly equal length [21]. Both proteins are encoded from the RD1 region of the *M. tuberculosis* genome, a region that has been found to be absent in the non-pathogenic strains of mycobacteria, esp. BCG [19]. Numerous structural and biochemical studies on this Esat6∶Cfp10 complex bear out their all alpha-helical tertiary fold, the interaction itself a result of hydrophobic-hydrophobic interactions between the two partners spread over an 1800 angstrom wide surface [22]. Although these proteins are not crucial for the survival of *M. tuberculosis*, their absence potently reduces, in many cases abrogates, the pathogenesis of the bacteria [18], [19], [23], [24]. More recently, it has been proposed that the 1∶1 complex dissociates post secretion, thus leaving Esat6 free to perform its functions, among which also include induction of host apoptosis [25], [26].

Our aim was to identify any host proteins that interact with free Esat6 either in a macrophage or in the extracellular matrix, once it has dissociated from its complex with Cfp10. In this context, we chose to screen the human lung proteins against Esat6, given that the primary site of infection is the human lung [7]. Herein, we report the identification of peptides that strongly bind Esat6 and, separately, appreciably affect the growth of the pathogen. None among these peptides were complete human proteins (and could thus be classified as inhibitors in their own entities); one was a portion of the mitochondrial Cox-3 protein, while the other two were formed as a result of aberrant ligation products during cDNA library construction. Nonetheless, the genes coding for all three peptides were sequenced and their integrity confirmed. A striking feature common to all three peptides was the presence of a high percentage of phenylalanine residues. One of the peptides, Hcl1, was constitutively expressed inside mycobacterium and its effects on the bacterial host investigated.

## Results

### Isolation of Peptides That Bind Esat6

**Figure 1:**
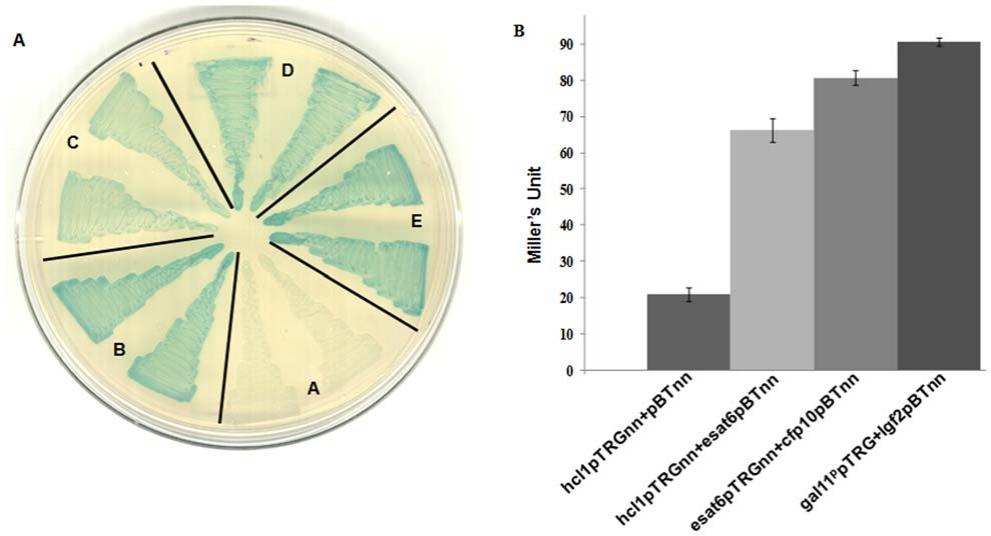
Interaction of Esat6 with human cDNA library (HCL) clones. (A) BacterioMatch two-hybrid reporter strain was co-transformed with (A) hcl1pTRGnn+pBTnn (negative control); (B) hcl1pTRGnn+esat6pBTnn; (C) hcl2pTRGnn+esat6pBTnn; (D) hcl3pTRGnn+esat6pBTnn; and (E) gal11^p^pTRG+lgf2pBT (positive control). Two individual colonies from each co-transformant were patched on X-gal indicator plates. (B) Confirmation of the *in vivo* interaction between Hcl1 and Esat6 using the liquid β-galactosidase assay. Assays were performed in duplicates with appropriate controls. Mean±s.d values are displayed here. A similar pattern was observed each time the experiment was repeated.

In order to search for host interacting partners of Esat6, bacterial two-hybrid reporter strain was co-transformed with esat6pBTnn and human lung cDNA library cloned in pTRG. We isolated three distinctly blue-coloured colonies, the colour being an indication that *in vivo* interaction had occurred. Employing repeated rounds of plasmid segregations, co-transformations, and in two cases PCR-amplification of the host gene followed by its re-ligation with the pTRG plasmid, we were able to confirm the interaction of the isolated host clones with Esat6 (Fig. 1A). To further validate the interaction, we performed liquid β-galactosidase assays and found that one of the host genes, named Hcl1, was a much stronger binder of Esat6 compared with the other two (Hcl2 and Hcl3; Fig. 1B). When sequenced, Hcl1 indicated an open reading frame (ORF) corresponding to a 27 amino acid long peptide (rich in phenylalanine) (Table 1). Interestingly, the ORF itself was a result of an incorrectly placed adaptor during the cDNA library construction, when the library members were being ligated to the recipient pTRG plasmid. A BLAST search showed that Hcl1 protein shared no homology with any known protein in the available databases.

**Table 1:** Primary amino acid sequences and characteristics of the peptide binders that interact with Esat6.

peptide	sequence	aa	MW	pI	% hydr.
Hcl1	AARIRHEGELVSS**FFFFFF**IENK**F**NDY *	27	3.3	5.5	41
Hcl2	AAHEGESTYQGHHTPPVQKGLRYGIIL**F**ITSEV**FFF**AG**FF** *	40	4.5	6.2	33
Hcl3	AARIRIEGTSLE**FFFFFF**PKKATLLMSCSSVH *	32	3.7	9.3	41

Phenylalanine residues are shown in bold.

**Footnotes:** aa: amino acids; MW: molecular weight; pI: isoelectric point; % hydr.: percentage hydrophobic content.

* stop codon.

Since Esat6 from *M. smegmatis* (MSMEG_0066) and Esat6 from *M. tuberculosis* (*Rv3875*) share 70% identity, we decided to explore if Hcl1 could bind *M. smegmatis* Esat6 as well. Indeed, in an *in vivo* bacterial two-hybrid experiment, we found that Hcl1 interacted with *M. smegmatis* Esat6 almost as potently as it did with *M. tuberculosis* (results not shown). The other two binder peptides, Hcl2 and Hcl3 (Table 1) also displayed an unusually high number of the Phenylalanine residues. While Hcl3, like Hcl1, was also formed form an aberrant adapter ligation, analysis of Hcl2 confirmed that it was part of a larger protein, the human Cox-3. To investigate whether the full-length human Cox-3 interacts with Esat6, we synthesized the wild type *cox-3* gene and cloned it in pTRGnn plasmid. We did not detect any interaction between the full-length Cox-3 and *M. tuberculosis* Esat6 (results not shown).

### Pull-Down Studies

**Figure 2:**
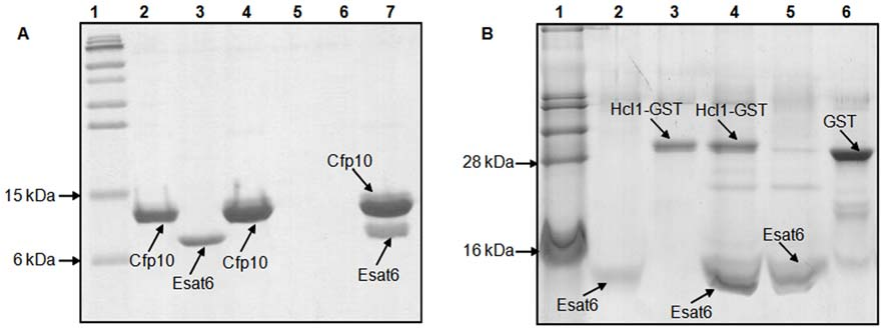
*In vitro* confirmation of the protein-protein interactions. (A) 16.5% Tricine-SDS polyacrylamide gel stained with coomassie blue showing interaction between purified Esat6 and Cfp10. Lane 1: pre-stained protein marker, Lane 2: purified His-tagged Cfp10; Lane 3: purified FLAG-tagged Esat6; Lane 4: 10 µg Cfp10 alone incubated with Ni-NTA agarose and eluted; Lane 5: 10 µg Esat6 alone incubated with 1% PVP blocked Ni-NTA agarose beads and eluted; Lane 6: empty; Lane 7: 10 µg Cfp10 incubated with Ni-NTA agarose beads and further incubated with 10 µg Esat6, and subsequently eluted. (B) 16.5% Tricine-SDS polyacrylamide gel stained with coomassie blue showing interaction between Esat6 and Hcl1. Lane 1: protein marker; Lane 2: purified His-tagged Esat6; Lane 3: purified Hcl1-GST; Lane 4: 5 µg Esat6 incubated with Ni-NTA agarose beads and further incubated with 5 µg Hcl1-GST, and subsequently eluted; Lane 5: beads initially bound with Esat6 and further incubated with purified GST protein; Lane 6: purified GST protein.

To further validate the interaction between Esat6 and Hcl1, *in vitro* pull-down assays were performed. For these assays, Esat6 was expressed as, separately, FLAG-tagged and His-tagged proteins, the proteins purified using DEAE-sepharose column under denaturing conditions and successfully resolubilized. To confirm the structural and conformational integrity of FLAG-Esat6 protein, we first studied its interaction with *M. tuberculosis* Cfp10. Cfp10 was expressed as containing an N-terminal 6X His-tag and purified as a soluble protein under native conditions using Ni-NTA agarose column. Additionally, Hcl1 was expressed and purified as a GST-fusion protein under native conditions using a glutathione-sepharose column. Equal amounts (10 µg) of the interacting proteins were used for pull-down experiments. We found that purified Esat6 interacted with both Cfp10 as well as Hcl1-GST proteins (Fig. 2A and 2B).

### Effect of Hcl1 on the Intracellular Survival of *M. tuberculosis*

**Figure 3:**
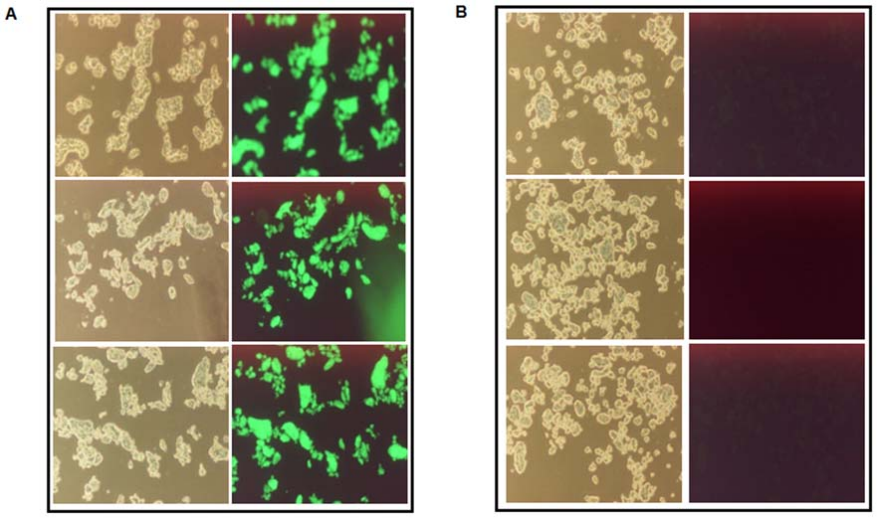
Expression of Hcl1 as a GFP fusion protein in *M. tuberculosis*. Three individual colonies each from mycobacteria harbouring either hcl1gfppVV16 (Mtb/GFP-Hcl1) or pVV16 (Mtb/pVV16) were grown till mid-log phase. Cells were examined under a fluorescent microscope. Panel A: Mtb/GFP-Hcl1 cells; Panel B: Mtb/pVV16 cells. First column in each panel shows bright field image of the sample.

Once it was confirmed that Hcl1 interacts with Esat6, we decided to investigate if the peptide has any effect on the intracellular survival of *M. tuberculosis* within macrophages. However, first, we cloned *hcl1* in another shuttle vector, gfppVV16 that has a C-terminal GFP-tag, to see if this peptide was expressed in mycobacteria in the first place. Cells were harvested from log phase cultures of *M. tuberculosis* H37Rv, harbouring hcl1gfppVV16 and examined under a fluorescent microscope. The green fluorescence, compared with its complete absence in the pVV16-harbouring control cells clearly demonstrated to us the expression of Hcl-GFP, also suggesting that peptides like Hcl1 could be synthesized in mycobacterium (Fig. 3). Furthermore, a BLAST scan of phenylalanine-rich proteins within *M. tuberculosis* revealed that mycobacteria are abundant with proteins rich in phenylalanine (results not shown).

**Figure 4:**
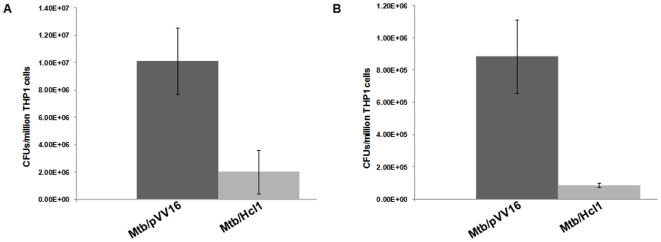
Effect of Hcl1 on survival of mycobacteria within macrophages. THP1 cells were infected with mycobacteria harbouring either hcl1pVV16 (Mtb/Hcl1) or pVV16 (Mtb/pVV16). The figure shows result of CFU counts, immediately after 4 hours of infection (A), called Day 0 and 24 hours post infection (B), called Day 1. Experiment was performed in triplicates and mean±s.d values are displayed here. A significant decline in the survival of mycobacteria carrying hcl1pVV16 was observed in comparison to the vector controls.

To study the effect of Hcl1 on the survival of mycobacteria within human macrophages, THP1 cells were infected with mycobacteria harboring either hcl1pVV16 (Mtb/Hcl1) or pVV16 (Mtb/pVV16) at an MOI of 10 for 4 hours at 37°C in an atmosphere of 5% CO_2_. Infected THP1 cells were harvested either immediately after infection (0 hours) or after 24 hours. THP1 cells were lysed and the mycobacteria plated on Middlebrook 7H11 agar plates for colony forming unit (CFU) analysis. Mycobacterial colonies were counted and mean±s.d values from one experiment were plotted against time (in hours). Similar pattern was observed each time the experiment was repeated. A significant reduction in the mycobacterial CFUs was observed in the sets where THP1 cells were infected with Mtb/Hcl1, in comparison to where they were infected with Mtb/pVV16 (Fig. 4)–approximately a 5-fold reduction in the number of colonies obtained from mycobacteria containing hcl1pVV16 in comparison to those harbouring just the vector control, pVV16, immediately after infection (Day 0, Fig. 4A). Although there was a significant decrease in colony count of mycobacteria one day after infection (Day 1), a more pronounced, approximately 10-fold reduction in CFUs was observed in mycobacteria expressing Hcl1, in comparison to the vector controls (Fig. 4B).

### Effect of Hcl1 on the In Vitro Growth of *M. tuberculosis*

**Figure 5:**
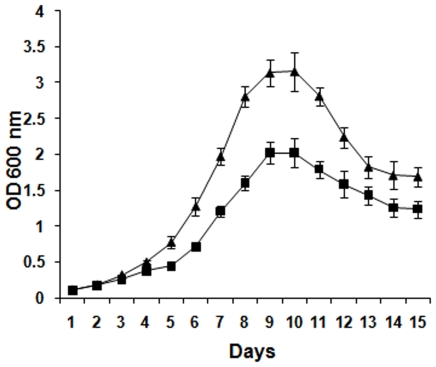
Effect of Hcl1 on the growth of *M. tuberculosis*. Three individual colonies each from mycobacteria harbouring either hcl1pVV16 (Mtb/Hcl1) or pVV16 (Mtb/pVV16) were picked for growth curve analysis. Optical density of each culture was measured at 600 nm. Mean±s.d values are plotted against time (in Days). A similar growth curve pattern was observed each time the experiment was repeated. Triangular data points represent OD_600_ values of Mtb/pVV16 samples; Squares represent OD_600_ values of Mtb/Hcl1 samples.

To study the effect of Hcl1 on general growth of mycobacteria, mycobacterial cells were transformed with either hcl1pVV16 (Mtb/Hcl1) or just the vector control, pVV16 (Mtb/pVV16). Three colonies from each transformant were picked for growth curve analysis. Optical density of each culture was measured after every 24 hours and plotted against time (in days) using mean±s.d (standard deviation) values from one experiment. Similar pattern was observed each time the experiment was repeated. It was found that the mycobacteria harbouring hcl1pVV16 showed a significant retardation in growth when compared with the vector controls (Fig. 5). The difference became evident as soon as the cells entered log phase and lasted till the culture reached stationary phase.

### Effect of Hcl1 on the Cell Morphology of *M. tuberculosis*

**Figure 6:**
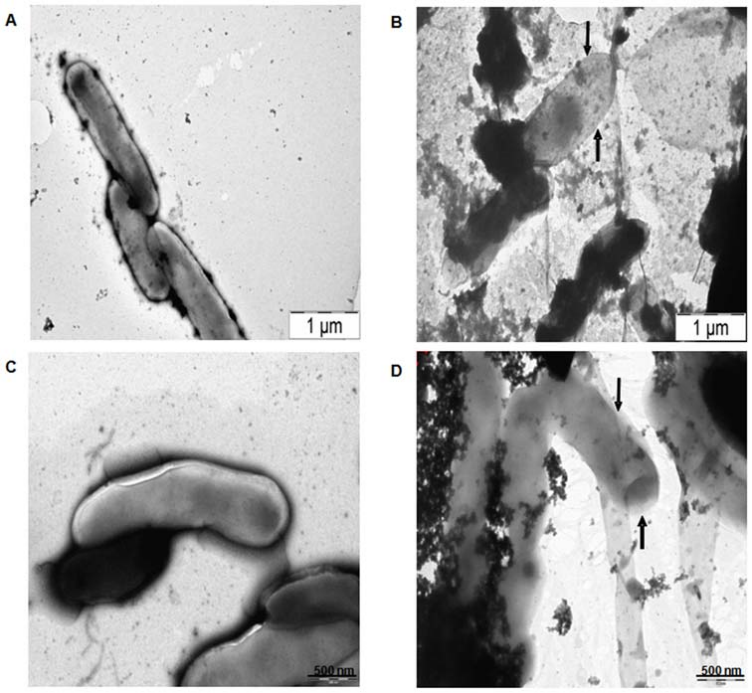
Effect of Hcl1 on cell shape and morphology of *M. tuberculosis*. Transmission electron micrographs of mycobacterial cells harbouring either pVV16 (Mtb/pVV16) or hcl1pVV16 (Mtb/Hcl1). Arrow heads in Panel B and D show the difference in cell morphology of mycobacteria containing Hcl1 when compared with the control (see Panel A and C).

Taking into consideration the effect of Hcl1 on the growth and intracellular survival of mycobacteria, we decided to investigate whether Hcl1 has any effect on cell shape and morphology. Electron micrographs of *M. tuberculosis* H37Rv cells harbouring either hcl1pVV16 or pVV16 alone were compared (Fig. 6). Cells harbouring hcl1pVV16 showed an extensive shedding of extracellular matrix from the surface (Fig. 6B and 6D). These cells with altered morphology seemed to be devoid of cell coat and appeared bare.

### Effect of Hcl1 on Global Gene Profile of *M. tuberculosis*

**Figure 7:**
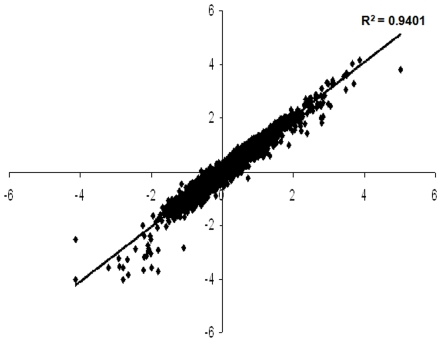
Scatter plot of average Z score values from treated (Mtb/Hcl1) and control (Mtb/pVV16) samples obtained after Z score transformation. Data points represent the average Z score values of genes obtained from six arrays.

Having seen the effect of Hcl1 on *M. tuberculosis* growth and cell morphology, we next performed microarray experiments to further explore the effects of Hcl1 on mycobacterial cells. Microarray experiments were performed with three biological replicates and four on-chip replicates. In order to identify genes that were differentially regulated in presence of Hcl1, we compared gene expression profiles of mycobacteria harbouring either hcl1pVV16 or pVV16 alone. Total RNA was isolated from log phase cultures of mycobacteria containing either hcl1pVV16 or pVV16, labelled with Cy3/Cy5 dyes and hybridized to microarray slides containing 4750 oligonucleotides (70-mer), each representing a unique *M. tuberculosis* H37Rv gene. Raw intensity values were transferred to excel spread sheet for analysis using the Z score method as described previously [29]. Raw intensity data was normalized using Z score transformation that produced Z score values for all the genes in each array (Fig. 7). The difference in average Z score values for all the genes in treated (Mtb/Hcl1) and control (Mtb/pVV16) was used to calculate the Z ratio. Genes having a Z ratio >1.5 and p value <0.05 were considered significant.

We found differential expression of 176 genes, of which 38 were found to be significantly upregulated and 138 genes significantly downregulated (Please see Supplementary Table S1). Of the genes that were upregulated, there were 7 genes coding for hypothetical proteins of unknown function, 6 conserved transmembrane proteins, 3 belonging to PE/PPE_PGRS family, 2 membrane transporters–one among them being Rv3877, part of Esx-1 secretion system, 2 transposases, 1 transcriptional regulator, 16 involved in various metabolic processes, and a protein kinase (*pknG*). Genes that were downregulated contain 84 genes coding for hypothetical proteins of unknown function, 22 genes involved in DNA replication, transcription, and translation, 4 belonging to PE/PPE_PGRS family, 20 genes involved in various metabolic pathways including cell wall synthesis, 2 genes coding for conserved transmembrane proteins of unknown function, one heat shock protein (*hspX*), 3 genes coding for putative secretory proteins: Rv0559c, EsxH, and EsxG, one twin-arginine translocase protein A (*tatA*), and a putative protein transporter, *secE2*.

**Figure 8:**
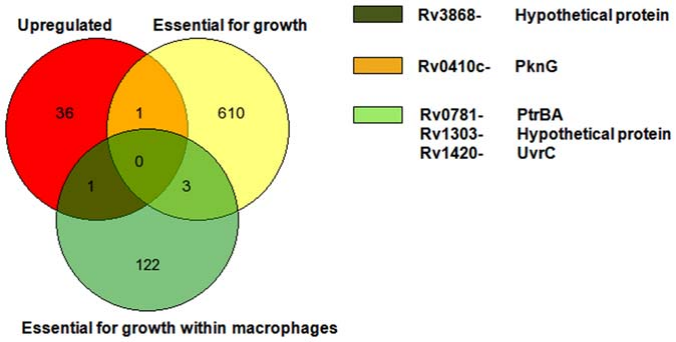
Comparison with Rubin's lists of genes that were found to be upregulated in microarray experiments. Red circle shows the genes upregulated; yellow and green circles show the genes essential for *in vitro* growth and for survival of mycobacteria within macrophages, respectively.

**Figure 9:**
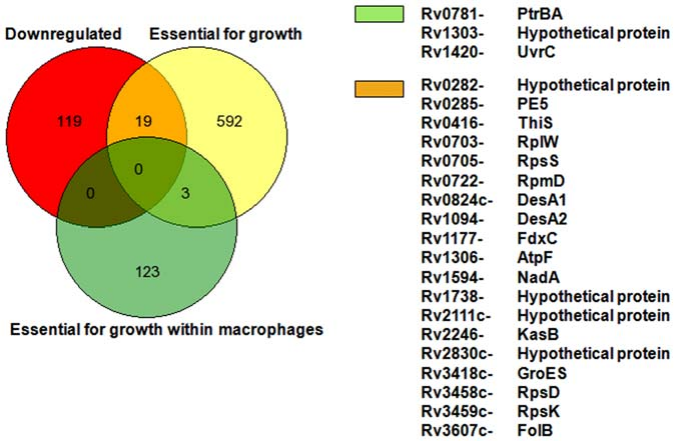
Comparison with Rubin's lists of genes that were found to be downregulated in microarray experiments. Red circle shows the genes upregulated; yellow and green circles show the genes essential for *in vitro* growth and for survival of mycobacteria within macrophages, respectively.

The differentially expressed genes were compared with the Rubin's list of essential genes for *in vitro* growth and growth within macrophages [31], [32]. We found one gene (*pknG*) that was found to be upregulated in our microarray experiment to be an essential gene required for *in vitro* growth of *M. tuberculosis* according to Rubin and co-workers (Fig. 8). Another gene, Rv3868, coding for a hypothetical protein, that was found to be upregulated has been shown to be essential for the growth of mycobacteria within macrophages (Fig. 8). Similarly, 19 genes that were downregulated in our experiments were found to be essential for *in vitro* growth of mycobacteria according to Rubin and co-workers (Fig. 9); some of these genes are involved in various metabolic processes including cell wall synthesis.

### Quantitative PCR

**Figure 10:**
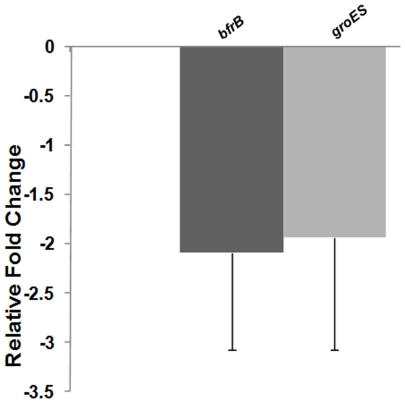
Relative fold change in the transcript levels of *bfrB* and *groES* genes. Real-time PCR was done using SYBR Green dye. Both genes were found to be significantly downregulated (∼2-fold) in Mtb/Hcl1cells when compared with the mycobacteria harbouring only pVV16. Experiment was performed using two biological replicates and in duplicates.

To validate our microarray experiments, we carried out real time PCR analysis of some of the identified genes. The genes chosen for such analysis were *groES* (co-chaperonin) and *bfrB* (bacterioferritin). The 16S rRNA gene was used as an internal control to compare the relative expression levels of these genes between treated (Mtb/Hcl1) and control (Mtb/pVV16) samples. Both the genes showed approximately 2-fold reduction in Mtb/Hcl1 samples in comparison to Mtb/pVV16 cells (Fig. 10).

## Discussion

In this study, we report isolation and characterization of phenylalanine-rich peptides that bind Esat6 protein of *M. tuberculosis*. Our aim was to study host-pathogen protein-protein interactions. For this purpose, we screened human lung cDNA libraries for potential interacting partners of the *M. tuberculosis* secretory protein Esat6. Using bacterial two hybrid system, we were able to isolate three strong binders against Esat6. Two among them, named Hcl1 and Hcl3, when sequenced, showed that their ORFs had resulted from incorrectly placed adaptors during the cDNA library construction, while the third, named Hcl2, was a fragment of human Cox-3 protein. Table 1 shows the three isolated peptides and their primary sequence characteristics. Without exception, they are hydrophobic in nature, possessing at least six to seven phenylalanine residues. While Hcl1 and Hcl3 display no homology to any know protein in the database–by virtue of the manner in which their corresponding ORFs have been formed–Hcl2 on the other hand is a fragment of the 260 aa long human Cox-3 protein, that, we showed, does not itself interact with Esat6. Human Cox-3 shares a very high degree of homology with other Cox-3 proteins, and prominently with the bovine counterpart, where the Hcl2 region is 86% identical to the human Cox-3. Interestingly, Hcl1 and Hcl3 display an all-helical fold, as seen in their threading models (results not shown), while Hcl2 fragment is also all-helical in the bovine Cox-3 protein (pdb code 2EIJ). We showed that the full-length human Cox-3 does not interact with Esat6, while Hcl2 fragment in isolation does. A closer look at the bovine Cox-3 crystal structure shows that Hcl2 forms part of a seven-bundle helix that is the Cox-3 protein; further, Hcl2 is partially embedded within the whole structure. It should be noted that both, Esat6 and Cfp10 are all-helical in character and their interaction is principally a hydrophobic one [22]. Additionally, peptides and proteins rich in phenylalanine have been seen to adopt helical folds, largely to do with the aromatic ring stacking [33].

That Esat6 plays an important role in *M. tuberculosis* virulence is well studied [17]–[20]. We decided to investigate the effect of Hcl1 binding to Esat6 and consequently its outcome on the survival of mycobacteria within macrophages. We noticed a significant decline in the survival of mycobacteria expressing Hcl1 within macrophages as indicated by the mycobacterial colony count post infection. Having studied the binding of Esat6 and Hcl1, both *in vitro* and in two-hybrid experiments, it was rational to assume that the observed effect could be because of this interaction resulting in the sequestration of Esat6. Esat6 and Cfp10 are part of the same operon, are co-transcribed and secreted as a tight 1∶1 complex by the Esx-1 secretion system [34], [21]. It is possible that the binding of Hcl1 to Esat6 poses steric hindrance for the interaction between Esat6 and Cfp10. A second possibility is that the Hcl1-Esat6 complex, when formed, interferes with the normal functioning of Esat6. Another interesting observation made during this study was that THP1 cells infected with mycobacteria containing pVV16 alone, showed extensive cell death as indicated by the size of the cell pellet obtained after the cells were harvested post-infection, in comparison to the THP1 cells infected with mycobacteria containing Hcl1. Live *M. tuberculosis* bacilli and Esat6 as such are known to induce apoptosis in macrophages [26]. It is possible that binding of Hcl1 to Esat6 or to some other molecule(s) inside the cell inhibits this process, although at present there is little proof to ascertain this conjecture and studies are currently underway to ascertain the possibility of the above-mentioned scenarios.

Next, we studied the effect of Hcl1 on the *in vitro* growth of mycobacteria. We found that mycobacteria containing Hcl1 showed a significant retardation in growth when compared to the controls. We were initially intrigued by this finding, since Esat6 is not known to play a defined role during the *in vitro* growth of mycobacteria [35]. Seeing the effect of Hcl1 on *in vitro* growth, we then checked its effect on cell morphology. We found that mycobacteria containing Hcl1 displayed an altered phenotype (Fig. 6). These cells showed extensive shedding of extracellular matrix and appeared bare.

We performed microarray experiments to further study the effect of Hcl1 on mycobacteria. We found a differential expression of 176 genes, of which 38 genes were significantly upregulated and 138 genes significantly downregulated. Using the elegant technique of transposon-based mutagenesis, Rubin and coworkers have identified sets of genes essential for *in vitro* growth of *M. tuberculosis* as well as for survival within macrophages [31], [32]. We compared the genes differentially regulated in our microarray experiment with Rubin's list of genes essential for *in vitro* growth and for growth within macrophages. On comparison, we found that, of the upregulated genes, *pknG* is essential for *in vitro* growth of mycobacteria, and another gene, Rv3868 (coding for a hypothetical protein), essential for survival of mycobacteria within macrophages. Similarly, 19 genes that were downregulated in our microarray experiment were found to be common to Rubin's list of genes essential for *in vitro* growth of mycobacteria. Among these were two genes for putative fatty acid desaturases (*desA1* and *desA2*), four hypothetical proteins, five ribosomal proteins, PE_PGRS family protein (PE5), *atpF* (ATP synthase subunit B), *groES* (co-chaperonin), *folB* (probable dihydroneopterin aldolase), *nadA* (quinolinate synthetase), *fdxC* (probable ferredoxin), *thiS* (sulfur carrier protein), and *kasB* (β-ketoacyl ACP synthase).

Mycobacteria contain a thick protective cell wall chiefly consisting of an extensively modified fatty acid complex, called mycolic acid [36]. It has been suggested that acyl desaturases play a role in the biosynthesis of mycolic acids by introducing position-specific double bonds that can subsequently be modified [37], [38]. Considering the debilitating effect of Hcl1 on cell surface of mycobacteria, it is tempting to speculate that a downregulation of *desA1* and *desA2* could affect mycolic acid biosynthesis and hence the cell wall integrity. Similarly, it has been shown that a deletion of *kasB* (involved in the biosynthesis of meromycolic acids) causes a change in mycobacterial colony morphology and loss of acid-fastness [39]. This again correlates well with the observed alteration in cell envelope of mycobacteria in this study. However, the effect may not exactly be of the same type as in case of the *ΔkasB* strain, for in our case, it is not a deletion *kasB* but mere downregulation. Also, in a recent study, *kasB* and *desA2* were shown to be significantly downregulated in mycobacteria treated with vancomycin [40]. The authors suggest that under conditions of growth arrest, bacteria slow down the synthesis of energy expensive cell wall components. *M. tuberculosis* contains one *cpn10* (*groES*) gene present upstream of *cpn60.1* (*groEL1*), which codes for a 10 kDa partner protein, Cpn10 [41]. Interestingly, it has been demonstrated that in *M. smegmatis*, GroEL1 interacts with KasA and modulates mycolic acid synthesis during biofilm formation, and a *groEL1* mutant of *M. smegmatis* showed significant reduction in the levels of KasA and KasB [42]. Since *M. tuberculosis* encodes a similar groEL protein and Cpn10 as a helper protein, it is possible that downregulation of *cpn10* could affect the functioning of Cpn60.1 during mycolic acid synthesis.

Other repressed genes, namely, *thiS*, *fdxC*, *nadA*, *atpF*, and *folB* code for proteins known to be involved in various metabolic pathways like siderophore biosynthesis, electron transport, NAD synthesis, ATP synthesis, and folate biosynthesis, respectively. It seems likely that all genes that were differentially regulated are directly/indirectly responsible for the observed effects of Hcl1 on growth and survival of mycobacteria. These findings suggest that Hcl1 is either working as a general antimicrobial peptide or interacting with some other molecule(s) inside the cell and hence interfering with normal cellular processes. As has already been mentioned, a threading-based modelling predicted a helical structure for Hcl1, a common feature of many antimicrobial peptides, and many such peptides have been found to be rich in phenylalanine residues [43]. However, whereas Hcl1 showed adverse effects on the growth of *M. tuberculosis*, it had no visible effect on the growth of *Escherichia coli* (*E. coli*), strengthening our belief that Hcl1 is interacting with molecule(s) specific to the mycobacterial cell.

In conclusion, this study demonstrates that Hcl1, a small peptide unusually rich in phenylalanine residues, affects the growth and pathogenesis of *M. tuberculosis*. What remains to be seen is whether the presence of Hcl1 in mycobacteria hinders Esat6 secretion. This could be assessed by comparing the levels of Esat6 protein in the culture filtrate of mycobacteria with and without Hcl1. Also, a pull-down assay from mycobacterial cell lysate is likely to shed some light on what might be many mycobacterial interacting partners of Hcl1. The localization of Esat6, in the presence and absence of Hcl1, within infected macrophages would also, we believe, shed new light on the possibility that Esat6 is involved in a hitherto unknown mechanism that helps the mycobacteria evade the host immune apparatus to a large extent. Finally, we hope that these studies might provide important clues regarding the mechanism of action of Hcl1 and other such phenylalanine-rich peptides and the information thus obtained could be further utilized in designing/improving peptide-based inhibitors against *M. tuberculosis*.

## Materials and Methods

### Bacterial Two-Hybrid Studies

Bacteriomatch™ two-hybrid system vector kit and Bacteriomatch™ Reporter strain competent cells were purchased from Stratagene, USA. The Bacteriomatch™ kit was supplied with bacterial two-hybrid system plasmids pBT, pTRG and control plasmids pBT-LGF2 and pTRG-Gal11^p^. The human lung cDNA library (cloned in pTRG vector) was purchased from Stratagene, USA. The bait and target expression vectors were modified to have a unique *Sna*BI site in its MCS for blunt end clonings [8]. The full length gene for e*sat6* (Rv3875) was amplified from *M. tuberculosis* H37Rv genomic DNA using forward (5′-CCT ACG TAA TGA CAG AGC AGC AGT GGA A-3′) and reverse (5′-CCT ACG TAT GCG AAC ATC CCA GTG ACG T-3′) primers, and cloned in pBTnn vector. Also, the corresponding homologue of e*sat6* (*MSMEG_0066*) from *M. smegmatis* LR222 was amplified from genomic DNA using the forward (5′-CCG AAT TCT ACG TAA TGA CAG AAC AGG TAT GGA ATT TC-3′) and reverse primers (5′-CCG AAT TCT ACG TAG GCA AAC ATT CCC GTG ACG CCG GCC-3′) and cloned in pBTnn.

Reporter strain competent cells were co-transformed with equal amounts (250 ng each) of esat6pBTnn and human lung cDNA library and plated on X-Gal indicator plates containing kanamycin (50 µg/ml), chloramphenicol (30 µg/ml), tetracycline (12.5 µg/ml), X-Gal (80 µg/ml), IPTG (25 µM), and X-Gal inhibitor (200 µM). Plasmids pBT-LGF2 (λcI-LGF2 fusion) and pTRG-Gal11^p^ (RNAPα subunit-Gal11^p^ fusion) provided the positive controls, whereas empty pBTnn and pTRGnn plasmids served as negative controls for all *in vivo* interaction studies. Positive interactions were judged by the blue colour of the colonies obtained and further verified by repeated clonings and cotransformations. The interactions were further verified by performing liquid β-galactosidase assay as described earlier [27].

### Cloning of hcl1, Esat6, and Cfp10 for Protein Expression

Forward (5′-AAG GAT CCT ACG TAA GAA TTC GGC ACG AG-3′) and reverse (5′-AAG GAT CCT ACG TAA TAA TCA TTA AAC TT-3′) primers were used to amplify the ORF corresponding to Hcl1 protein from the original lung cDNA library clone. The PCR product was digested and cloned in *Sna*BI site in pGEX4T3nn vector [8] resulting in a fusion protein with a C-terminal GST tag. The esat6pBTnn plasmid DNA was digested with *Not*I/*Bgl*II and the resulting 346 bp fragment was cloned in *Not*I/*Bam*HI-digested pFLAGCMV6a vector to obtain esat6pFLAGCMV6a. The esat6pFLAGCMV6a plasmid DNA was digested with *Nco*I/*Eco*RI and a 359 bp fragment was cloned in *Nco*I/*Eco*RI-cut pET28a (Novagen) vector. The clone, flagesat6pET28a, resulted in expression of Esat6 as a fusion protein with an N-terminal FLAG-tag but no His-tag. The ORF corresponding to Cfp10 protein was PCR amplified from *M. tuberculosis* H37Rv genomic DNA using forward (5′- CCT ACG TAA TGG CAG AGA TGA AGA CCG A-3′) and reverse (5′- CCT ACG TAA GTA CTG AAG CCC ATT TGC GAG GAC A-3′) primers. The PCR product was digested with *Sna*BI and cloned in *Sna*BI-cut pBADHisAnn vector. The clone, cfp10pBADHisAnn, resulted in expression of Cfp10 as fusion protein with a C-terminal 6x His-tag. Similarly, for the expression of Esat6 with a C-terminal 6X His-tag, the ORF was amplified as described above from H37Rv genomic DNA and cloned in pBADHisAnn vector.

### Protein Expression and Purification of Hcl1-GST

*E. coli* BL21 (DE3) (Novagen) cells harbouring hcl1pGex4T3nn were grown till mid-log phase and induced with 1 mM IPTG for 3 hours with constant shaking at 37°C. Cells were harvested and the pellet was washed with PBS (40 mM K_2_HPO_4_, 10 mM KH_2_PO_4_, 150 mM NaCl, pH 7.2). Cell pellet was resuspended in (1% of the original culture volume) lysis buffer (100 mM EDTA, 1 mM PMSF, 0.5 mM DTT in PBS) and sonicated till a clear lysate was obtained. Triton X-100 was added to a final concentration of 1% and the tubes were incubated at 4°C for 30 minutes with constant shaking. Lysate was then centrifuged and the clear supernatant was transferred to fresh tubes. Glutathione-agarose beads (Amersham) were washed thrice with PBS. Desired amount of glutathione-agarose beads were added to the supernatant and incubated at 4°C for 3 hours with constant shaking. Flow-through was collected and beads were washed thrice with PBS containing 1% Triton X-100 followed by a single wash of 50 volume of TN buffer (50 mM tris-Cl, 150 mM NaCl, pH 7.5). Resin bound proteins were eluted using elution buffer (10 mM reduced glutathione in 50 mM tris-Cl, pH 8.0) and analyzed on SDS-PAGE. Purified protein was dialyzed against PBS, pH 7.5, quantified and stored at −20°C in aliquots.

### Protein Expression and Purification of Esat6

*E. coli* BL21 (DE3) cells harbouring flagesat6pET28a were grown till mid-log phase and induced with 1 mM IPTG for 3 hours with constant shaking at 37°C. Cells were harvested and the pellet was washed with PBS. Cell pellet was resuspended in lysis buffer (8 M urea in 40 mM phosphate buffer, pH 7.5) and sonicated till a clear lysate was obtained. The lysate was centrifuged and the supernatant was transferred to a fresh tube. The supernatant was mixed with DEAE-sepharose (Amersham) and incubated at room temperature for 1.5 hours with constant shaking. Beads were washed thrice with lysis buffer and the resin bound proteins were eluted using a gradient of NaCl from 0 M to 1 M, prepared in lysis buffer. The gradient elution was performed using AKTA FPLC (Amersham) system and the eluted fractions were analyzed on SDS-PAGE. For purification of His-tagged Esat6, log phase culture of *E. coli* TOP10 (Invitrogen) cells containing esat6pBADHisAnn were induced with 0.02% L-arabinose for 3 hours at 37°C. Cells were pelleted and washed with PBS. Pellet was resuspended in lysis buffer (100 mM NaH_2_PO_4_, 10 mM tris-Cl, pH 8.0 and sonicated. Lysate was cleared by centrifugation and pellet thus obtained was solubilized in lysis buffer containing 8 M urea, 10 mM imidazole, and 1% Triton X-100. Solution was centrifuged and supernatant was loaded on a Ni-NTA agarose (Qiagen) column. Beads were washed once with buffer B and thrice with buffer C (buffer B containing 20 mM imidazole). Finally, protein was eluted using elution buffer (100 mM NaH_2_PO_4_, 10 mM tris-Cl, 8 M urea, 350 mM imidazole, pH 8.0). Purified protein was refolded by sequentially and slowly dialyzing it against PBS with decreasing concentration of urea. Protein was concentrated using Amicon® Ultra (Millipore) column, quantified and stored at −20°C in aliquots.

### Protein Expression and Purification of Cfp10

*E. coli* TOP10 cells harbouring cfp10pBADHisAnn were grown till mid-log phase and induced with 0.02% L-arabinose for 3 hours at 37°C. Cells were pelleted and resuspended in lysis buffer (50 mM NaH_2_PO_4_, 300 mM NaCl, and 10 mM imidazole, pH 8.0). Lysozyme was added to a final concentration of 1 mg/ml and tubes were incubated at 4°C for 30 minutes with shaking. Samples were sonicated till a clear lysate was obtained. Triton X-100 was added to a final concentration of 1% and incubated at 4°C for 30 minutes with shaking. Lysate was centrifuged and the supernatant was loaded on a Ni-NTA agarose column. Beads were washed once with lysis buffer and thrice with wash buffer (50 mM NaH_2_PO_4_, 300 mM NaCl, and 20 mM imidazole, pH 8.0). Protein was eluted using elution buffer (50 mM NaH_2_PO_4_, 300 mM NaCl, and 300 mM imidazole, pH 8.0), dialyzed against PBS, quantified and stored at −20°C.

### In Vitro Protein-Protein Interaction

#### Esat6 and Cfp10

Ni-NTA agarose beads were mixed with 10 µg of purified Cfp10-His protein and incubated at 4°C with shaking for 1.5 hours. Beads were washed twice with 50 mM phosphate buffer containing 500 mM NaCl (wash buffer 1) and once with PBS containing 0.1% Tween-20 and 14 mM β-mercaptoethanol (wash buffer 2). Beads were then blocked with 1% polyvinyl pyrrolidone (PVP) for 1 hour. To this, 10 µg of purified FLAG-Esat6 protein was added and incubated at 4°C for 1.5 hours. In a control experiment, Ni-NTA agarose beads were blocked with 1% PVP and incubated with 10 µg of purified FLAG-Esat6 protein at 4°C for 1.5 hours. Finally, beads were extensively washed with wash buffer 1. Resin bound proteins were eluted by boiling the beads in 2X SDS loading dye (20% glycerol, 4% SDS, 125 mM tris-Cl, pH 6.8, and 4% β-ME) and resolved on 16.5% tricine-SDS polyacrylamide gel.

#### Esat6 and Hcl1

Purified His-tagged Esat6 protein (10 µg) was incubated with Ni-NTA agarose beads for 1.5 hours at 4°C. Beads were washed twice with buffer B (100 mM NaH_2_PO_4_, 10 mM tris-Cl, 20 mM imidazole, pH 7.4) followed by two washes with buffer B containing 30 mM imidazole and finally one wash with buffer C (30 mM imidazole in PBS, pH 7.4). To the beads, 10 µg purified Hcl1-GST protein was added and kept for binding at 4°C for 1.5 hours. In a control experiment, 10 µg purified GST protein was added to Esat6 bound Ni-NTA agarose beads. Beads were finally washed twice with buffer C and bound proteins were eluted by boiling the beads with 2X SDS loading dye and resolved on 16.5% tricine-SDS polyacrylamide gel.

### Construction of Expression Vectors

#### pVV16*Sna*BI (called pVV16) vector

The original pVV16 vector was modified to have a unique *Sna*BI site in its MCS for blunt-end clonings. Forward (5′-CCC CAT GGC ATA TGT ACG TAA TGA TTG ATG AGG CTC TCT TCG A-3′) and reverse (5′-CCC TCG AGA AGC TTT ACG TAG ACC TCC AGC AGC TCG CCT TC-3′) primers were designed to amplify a 599 bp fragment from frrpBTnn. Amplified PCR product was digested with *Nde*I*/Hind*III and cloned in *Nde*I/*Hind*III-digested pVV16 vector. The resulting clone, frrpVV16 was digested with *Sna*BI to remove the insert and self-ligated to generate, pVV16*Sna*BI. The gene corresponding to *hcl1* was amplified from original lung cDNA library clone using forward (5′-AAG GAT CCT ACG TAA GAA TTC GGC ACG AG-3′) and reverse (5′-AAG GAT CCT ACG TAA TAA TCA TTA AAC TT-3′) primers, digested with *Sna*BI and cloned in pVV16 to generate hcl1pVV16.

#### gfppVV16SnaBI (called gfppVV16) vector

Forward (5′-CCG AAT TCT ACG TAA TGG TGA GCA AGG GCG AGG AGC TG-3′) and Reverse (5′-CCG AAT TCA GTA CTT TAC TTG TAC AGC TCG TCC ATG CC-3′) primers were used to amplify 748 bp GFP gene using pGFPN1 (Clontech) as template. The PCR product was digested with *Sna*BI/*Sca*I and cloned in *Sna*BI-cut pVV16 vector to generate gfppVV16*Sna*BI. The resulting vector had a unique *Sna*BI site upstream of *gfp* gene. The gene corresponding to *hcl1* was amplified as described above and cloned in *Sna*BI-cut gfppVV16 to generate hcl1gfppVV16.

### Expression of Hcl1 in *M. tuberculosis*

Mycobacteria harbouring either hcl1gfppVV16 or pVV16 were grown till mid-log phase. Cells were harvested, washed with PBS and fixed by treating with a 2% paraformaldehyde solution for 1 hour at 4°C [28]. Cells were examined using advanced inverted microscope, ECLIPSE TE 2000-S (Nikon) for GFP fluorescence.

### Infection of Human Macrophages with *M. tuberculosis*

THP1 cells were grown in RPMI 1640 media supplemented with 10% fetal bovine serum. Cells were seeded in flat bottom culture flask at a confluence of around 80% and induced with 50 ng/ml phorbol-12-myristate-13-acetate (Sigma) for 12 hours. Activated THP1 cells were infected (MOI = 10) with *M. tuberculosis* H37Rv bacilli harbouring either hcl1pVV16 or pVV16, for 4 hours at 37°C, in an atmosphere of 5% CO_2_. Cells were harvested and washed with pre-warmed medium to remove extracellularly present bacteria. Additionally, cells were treated with gentamicin (Sigma, 50 µg/ml) for 2 hours at 4°C to kill extracellularly present bacteria. Finally, cells were washed, resuspended in PBS and lysed with 1% NP-40 by vortexing. Supernatant was diluted and plated on Middlebrook 7H11 agar (Difco™, BD) plates supplemented with 10% OADC (BBL™, BD), 100 µg/ml cycloheximide, 0.5% glycerol, and 25 µg/ml kanamycin. The plates were incubated at 37°C till the colonies appeared.

### Growth Curve Studies of *M. tuberculosis*

Mycobacteria harbouring hcl1pVV16 (Mtb/Hcl1) or pVV16 (Mtb/pVV16) were grown in 10 ml Middlebrook 7H9 broth at 37°C and 200 rpm till stationary phase was reached. Optical density of the culture was measured at 600 nm using Lambda 35 spectrometer (PerkinElmer) and equal numbers of cells from each culture were freshly inoculated in 50 ml flask containing fresh Middlebrook 7H9 broth. Cells were allowed to grow at 37°C and 200 rpm. Optical density of each culture was measured at 600 nm after every 24 hours.

### Transmission Electron Microscopy

*M. tuberculosis* H37Rv cells harbouring either hcl1pVV16 or pVV16 were grown (at 37°C with constant shaking) till mid-log phase in Middlebrook 7H9 broth (Difco™, BD), supplemented with 10% ADC (BBL™, BD), 0.05% Tween-80, 0.2% glycerol, and 25 µg/ml kanamycin. Cells were harvested, washed with PBS and fixed by treating with 2% paraformaldehyde solution for 1 hour at 4°C [28]. Fixed cells were allowed to adsorb on a 300 mesh copper grid and air dried. Samples were then stained with 1% uranyl acetate for 30 seconds and photographed using a FEI Tecnai 12 Electron Microscope.

### Total RNA Isolation from *M. tuberculosis*

Total RNA was isolated as described previously [8] with some modifications. Cells were harvested from exponentially growing mycobacteria harbouring either hcl1pVV16 (Mtb/Hcl1-treated sample) or pVV16 (Mtb/pVV16-control sample) and washed with 0.1% Tween 20. Pellet was resuspended in 400 µl TES buffer (50 mM Tris-Cl, pH 7.4, 10 mM EDTA, and 1% SDS). To this an equal volume of water-saturated phenol was added. Approximately an equal volume of acid-washed glass beads was added for efficient cell disruption. Tubes were vigorously vortexed and incubated at 65°C for 1 hour with intermittent vortexing after every 15 minutes. Finally, the tubes were chilled on ice for 5 minutes and centrifuged at 13,000 rpm for 15 minutes at 4°C. Aqueous phase was transferred to fresh tubes and extracted thrice with chloroform: isoamylalcohol (24∶1). RNA was precipitated with 0.1 volume of 3 M sodium acetate and 2.5 volume of chilled ethanol. For efficient precipitation of RNA, tubes were incubated at −80°C for 30 minutes and centrifuged at 13,000 rpm for 30 minutes at 4°C. RNA pellet was washed with 70% ethanol, dried and resuspended in 30 µl of DEPC-treated water. RNA was checked for quality on 1% denaturing agarose gel and purified using RNeasy purification kit (Qiagen) followed by on-column *DNase*I (0.7 U/µl) (Ambion) treatment for 30 minutes at room temperature, as described by the manufacturer. RNA was checked for quantity and quality on a NanoDrop™ ND-1000 spectrophotometer (Thermo Scientific).

### Labeling of cDNA with Cy Dyes

Equal amounts of RNA (9.0 µg) from both control (Mtb/pVV16) and treated (Mtb/Hcl1) samples were used for preparing labeled cDNA for microarray experiment. Total RNA was labeled with Cy3/Cy5-dUTP using CyScribe First-Strand cDNA labeling kit (Amersham) as described by the manufacturer. Labeled product was treated with *RNase*A (0.03 U/µl) (Fermentas) and *RNase*H (0.05 U/µl) (Fermentas) for 20 minutes at 37°C to remove the RNA. Labeled cDNA was purified and eluted using MinElute PCR Purification kit (Qiagen) and analyzed on a NanoDrop™ spectrophotometer for quantity and quality. Incorporation of Cy3 and Cy5 dye was determined by measuring the absorbance at 550 nm and 650 nm, respectively. Labeled cDNA was vacuum dried and stored at −20°C till further use.

### Microarray Slides Hybridization

*M. tuberculosis* H37Rv microarray slides (Version 4) were obtained from Pathogen Functional Genomics Resource Center (PFGRC), J. Craig Venter Institute (JCVI). The hybridization protocol used here is as described by The Institute for Genomic Research, SOP#8 (http://pfgrc.jcvi.org/index.php/microarray/protocols.html) with some minor modifications. The slides were incubated in pre-warmed (at 42°C) pre-hybridization buffer (5X SSC, 0.1% SDS, 1% BSA) and incubated at 42°C for 1 hour in 50 ml Falcon tubes. The slides were thoroughly rinsed with distilled water followed by isopropanol and quickly dried by centrifugation at 1,500 rpm for 15 minutes. The labeled cDNA probe was resuspended in 50 µl hybridization buffer (5X SSC, 40% formamide, 0.1% SDS, 0.1 mM DTT, 0.6 µg/ml salmon sperm DNA). This probe mixture was vortexed and heated at 95°C for 5 minutes, vortexed and then again heated at 95°C for another 5 minutes. The denatured probe mixture was then added to the slides and covered with a coverslip. The hybridization cassette (Corning) was sealed and submerged in a water bath set at 42°C for 20 hours. Post hybridization, coverslips were removed and slides were washed 5 times with pre-warmed (at 55°C) Low Stringency Wash buffer (2X SSC, 0.1% SDS, 0.1 mM DTT). Slides were then washed 5 times with Medium Stringency Wash buffer (0.1X SSC, 0.1% SDS, 0.1 mM DTT). Finally, slides were washed five times with High Stringency Wash buffer (0.1X SSC, 0.1 mM DTT) and thoroughly rinsed with distilled water. Slides were dried by centrifugation at 1,500 rpm for 15 minutes and stored under dark and dry conditions.

### Microarray Data Analysis

Slides were scanned using a dual laser ScanArray Gx Scanner (PerkinElmer) at best signal and least noise PMT gain. ScanArray Express (PerkinElmer) software was used to convert the hybridization signals into raw intensity values, which were then transferred to Microsoft Excel spreadsheet for further analysis. Data analysis was done using Z-score method described previously [29]. Raw intensity data for Cy3/Cy5 dyes (for both Mtb/pVV16-control as well as Mtb/Hcl1-treated samples) as ‘mean-background’ was extracted from six arrays and used for normalization. Raw intensity values were log_10_ transformed and average and standard deviation calculated for all the genes in each replicate and across all the arrays. Z-score values were calculated by subtracting the overall average intensity from log_10_ transformed raw intensity and dividing by measured standard deviation in a single experiment and for all replicates. Average of all the replicates for both treated (Mtb/Hcl1) and control (Mtb/pVV16) samples were used to calculate Z-Score difference (Treated-Control). Test of significance for changes in gene expression was measured by calculating the Z-ratios and p values in a standard T-test (two class unpaired, two tailed). Z-ratios were calculated by measuring the difference between the average Z-score of genes from treated samples (Mtb/Hcl1) and from control samples (Mtb/pVV16) and dividing them by the standard deviation of the difference. Genes having a Z-ratio >1.5 and p value <0.05 were considered significant.

The microarray data is MIAME compliant and has been deposited in a MIAME compliant database GEO (Accession No. GSE16618).

### Real Time PCR

5.0 µg RNA from both control (Mtb/pVV16) and treated (Mtb/Hcl1) samples was reverse transcribed using CyScribe First strand cDNA labeling kit (Amersham). Random nonamer primers were added to RNA sample in a total volume of 11 µl. This mixture was heated at 70°C for 5 minutes and then allowed to cool for 10 minutes at room temperature. Subsequently, 4 µl of 5X CyScribe buffer, 1 µl of 100 mM DTT, 1 µl of 10 mM dNTPs, and 1 µl of CyScribe reverse transcriptase were added in a total volume of 20 µl. Reaction mixture was incubated at 42°C for 1.5 hours. Reaction was stopped by inactivating the enzyme at 90°C for 10 minutes. Real time PCR was performed using IQ SYBR Green Supermix (BioRad) in a total reaction volume of 25 µl. The reaction mixture consisted of 0.4 µM of each primer, 12.5 µl of 2X IQ SYBR Green Supermix, and 1 µl of diluted cDNA template (equivalent to 8 ng RNA). Amplification was performed in a MiniOpticon (BioRad) thermocycler using following conditions: (i) an initial denaturation step of 10 minutes at 95°C; (ii) 40 cycles, each consisting of 30 seconds at 95°C, 30 seconds at 58°C, and 30 seconds at 72°C. Fluorescence was measured at the end of the elongation step during each cycle. A melt curve analysis was performed between 55°C and 95°C with an increment of 0.5°C per 10 seconds. PCR product was also checked on 1.7% agarose gel. Data was analyzed using comparative C_T_ method as described earlier [30]. 16S rRNA was used as an internal control gene for normalization.

## Supporting Information

Table S1 Differentially regulated genes in microarray experiments.
(0.05 MB XLS)
